# New-onset lone maternal atrial fibrillation: A case report

**DOI:** 10.1097/MD.0000000000019156

**Published:** 2020-02-14

**Authors:** Nusrat Batool Janjua, Suhaib Akhtar Birmani, Thomas McDonagh, Abdul Hameed, Matthew McKernan

**Affiliations:** a Cavan and Monaghan General Hospital, Co. Cavan. Ireland; b University Hospital, Galway; c Altnagalvin Area Hospital, Londonderry, UK; d Letterkenny University Hospital. Co. Donegal, Ireland.

**Keywords:** atrial fibrillation, electrical cardioversion, maternal, pregnancy

## Abstract

**Rationale:**

Atrial fibrillation (AF) is encountered rarely in pregnancy. Management of maternal AF is challenging as it poses a threat to both maternal and fetal well-being.

**Patient concerns:**

We report a case of a 35 weeks pregnant woman who presented in emergency with sudden-onset palpitations and mild shortness of breath with no personal/family history of cardiac diseases.

**Diagnoses:**

Patient's pulse was irregularly irregular with an average rate of 179 beats per minute. The obstetric examination was normal.

**Diagnosis:**

High-sensitive cardiac troponin T (hs-cTnT) was elevated. The 12 lead electrocardiogram (ECG) confirmed AF. The obstetric ultrasound, electronic fetal heart rate (EFHR) trace, and maternal echocardiography were normal.

**Interventions:**

The patient was admitted under joint cardiology and obstetric care and monitored with continuous telemetry. She was commenced on a therapeutic dose of low-molecular weight heparin (LMWH) and intravenous fluid. She received a single 200 Joule synchronized direct current (DC) shock under general anesthesia in operation theater, which reverted the rhythm back to normal. EFHR monitoring was normal pre- and post-DC cardioversion. We acknowledge the unwise use of therapeutic dose of LMWH before DC cardioversion (DCCV) because of a potential need for emergency cesarean delivery for maternal and/or fetal compromise.

**Outcome:**

The patient remained well and in sinus rhythm after cardioversion. She was discharged home the following day on Flecainide (anti-arrhythmic) and therapeutic dose of low molecular weight heparin (LMWH) and followed up in outpatient clinics frequently. She had a baby at term and received prophylactic LMWH for 10 days post-cesarean. She was discharged from cardiology clinic when she was 10 weeks postnatal, and Flecainide was discontinued.

**Lessons:**

We are reporting this case because of the rarity of the condition and successful use of DCCV for treating maternal AF. High-sensitive cardiac troponin T (hs-cTnT) level is a useful laboratory indicator to gauge the severity of AF in pregnancy. We emphasize to make the arrangements for EFHR monitoring and potential cesarean delivery and advocate cautious use of thromboprophylaxis while planning for electrical cardioversion (ECV) for maternal AF.

## Abstract 2

Supplemental Digital Content is available in the text

## 1 Introduction

Though atrial fibrillation (AF) is the commonest cardiac arrhythmia in adults, maternal AF is rare.^[1–3]^ It may occur de novo or be exacerbated by pregnancy. AF increases maternal and perinatal mortality and morbidity if not treated promptly, especially in patients with cardiac disease.^[4]^ electrical cardioversion (ECV) for AF has been reported as a safe procedure in pregnancy and its known complications include fetal bradycardia, need for emergency delivery, and preterm delivery.^[5]^ Thus, continuous electronic fetal monitoring before and after ECV and preparation for cesarean delivery are required.^[6]^ Clinical evidence for management of maternal AF is limited.^[4]^

Few cases of new-onset lone AF in pregnancy have been reported in the literature and obstetricians, emergency physicians, and cardiologists are less familiar with it.

## 2 Case presentation

### 2.1 Patient information

A 37-year- old woman, gravida 3, para 2 was referred to the emergency department of the hospital by her General Physician at 35 weeks of gestation with sudden-onset palpitations associated with mild shortness of breath. The patient denied any chest pain, dizziness, or pre-syncope. The patient had history of discharge from pediatric review for an asymptomatic childhood murmur. She had an echocardiograph for a systolic murmur at the aortic area during second trimester of her first pregnancy at 29 years of age, which was a normal study. She had 2 cesarean sections. There was no recent history of tobacco, drug, alcohol, lack of sleep, illness, caffeine, dehydration, or any other precipitating factors. There was no personal/family history of cardiac diseases.

### 2.2 Clinical findings

On examination, the patient was not obviously distressed and her pulse was irregularly irregular with an average rate of 179 beats per minute. The other vital signs included blood pressure 103/79 mm Hg, respiratory rate 16/minute and SpO_2_ 99% on room air. The Jugular venous pressure was not raised. There was no peripheral edema and both her calves were soft and nontender. Her chest was clear on auscultation. The abdomen was soft, nontender with a gravid uterus. Obstetric examination revealed a symphysio-fundal height corresponding to the dates and an average fetal heart rate of 146 beats/min.

### 2.3 Diagnostic assessment

**Figure 1:**
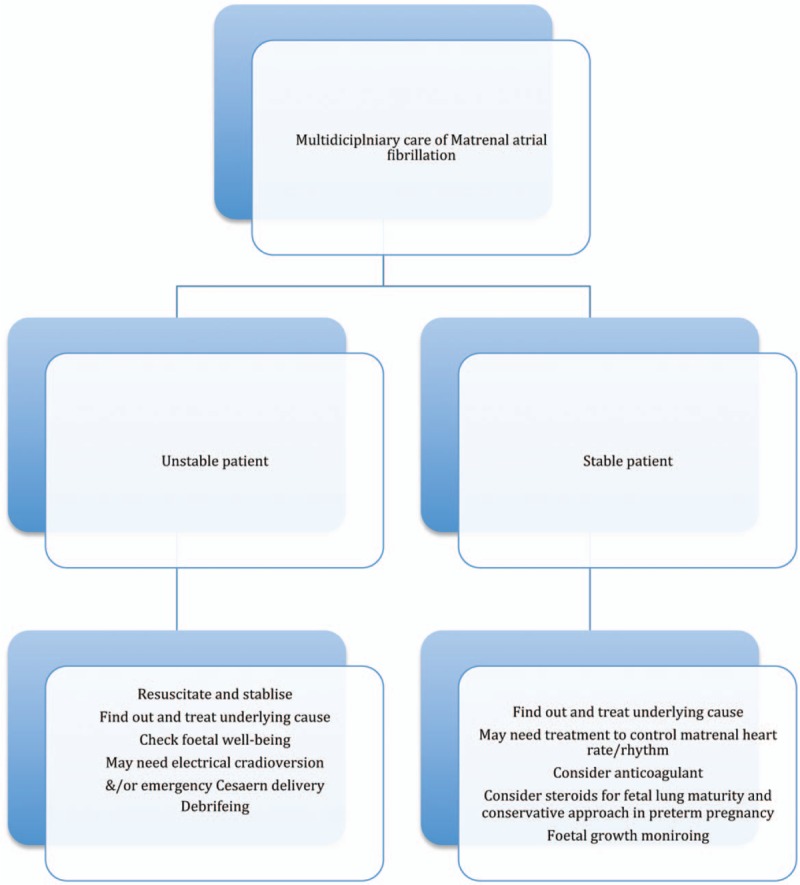
Management of Atrial Fibrillation in a Pregnant Female.

Blood tests showed normal hemoglobin, renal profile, electrolytes, thyroid function tests, and urine analysis. High-sensitive cardiac troponin T (hs-cTnT) was elevated at 72 ng/L (reference range: 0–9 ng/L). The 12 lead electrocardiogram (ECG) confirmed AF with a ventricular rate of 170 to 190 beats per minute (Fig. 1). An arterial blood gas test revealed that the pH was 7.43, partial pressure of carbon dioxide was 4.2 kPa, the partial pressure of oxygen was 7.4 kPa, and that of the bicarbonate was 20.3 mmol/L on room air. The obstetric ultrasound, EFHR trace, and maternal echocardiography were normal.

### 2.4 Therapeutic intervention

**Figure 2:**
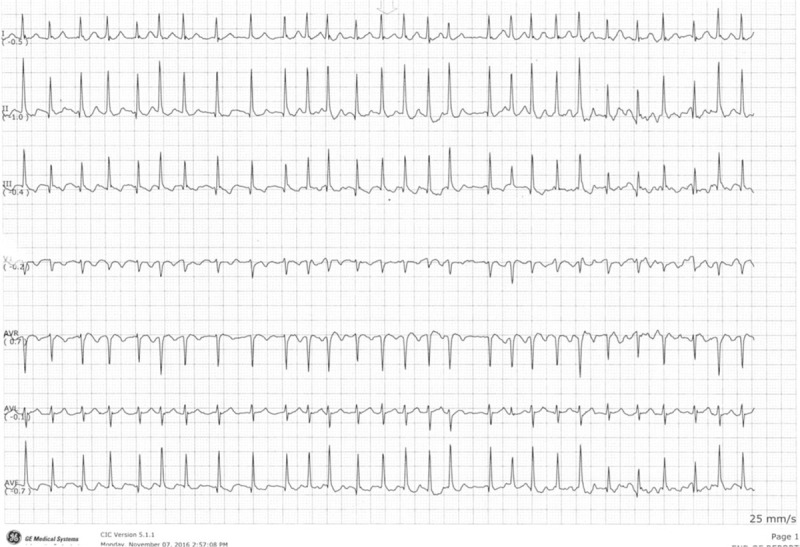
ECG (telemetry) showing fast AF.

**Figure 3:**
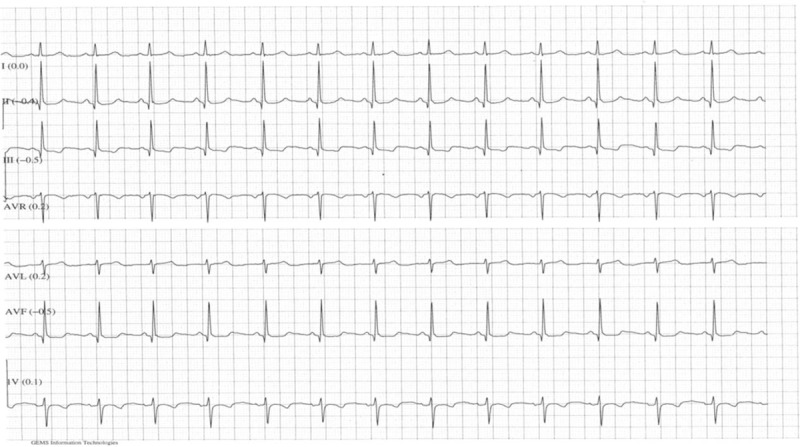
ECG after electrical cardioversion.

**Figure 4:**
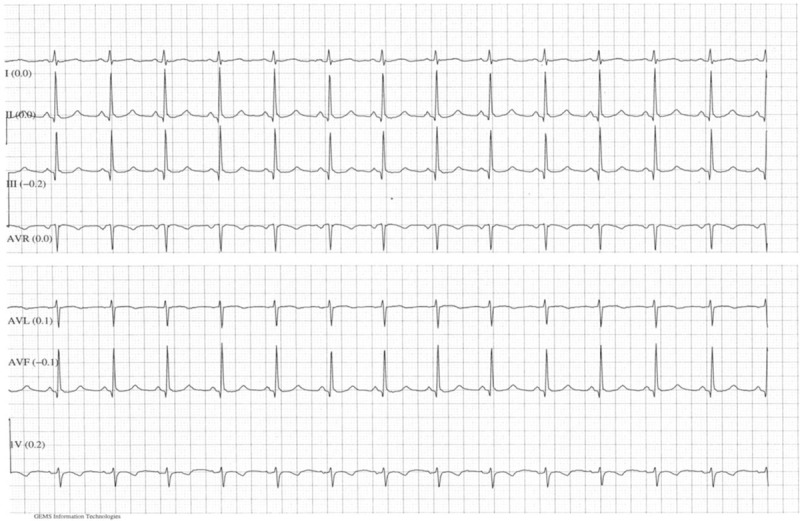
ECG after electrical cardioversion.

The patient was admitted under joint cardiology and obstetric care and monitored with continuous telemetry (Fig. 2). She was commenced on a therapeutic dose of low-molecular weight heparin (LMWH) and intravenous fluid. She received a single 200 Joule synchronized direct current (DC) shock under general anesthesia in operation theatre which reverted the rhythm back to normal. Fetal heart rate remained normal before, and after the procedure. The transabdominal obstetric ultrasound was also reassuring after the procedure. The obstetric team was prepared to perform emergency cesarean section in case of maternal and/or fetal compromise. She remained in sinus rhythm with a regular pulse rate between 88 and 96 beats/min and was monitored by telemetry overnight (Figs. 3 and 4). The antiarrhythmic Flecainide (50 mg tablet twice daily) was started. An echocardiograph performed the following day, showed normal ventricular size and function and no significant valve lesion, normal atrial dimensions, and no thrombus. Subsequent electronic fetal monitoring was also reassuring.

### 2.5 Follow-up and outcome

She was discharged the following day on a daily therapeutic dose of LMWH and Flecainide for the remainder of her pregnancy. The feto-maternal surveillance was satisfactory in subsequent frequent antenatal and cardiac visits. LMWH was stopped 2 days before her planned cesarean section and tubal ligation. She delivered a healthy baby girl at 39 weeks and 2 days with a birth weight of 3.34 kg. She had an uneventful surgery and postoperative recovery. She was discharged home on a prophylactic dose of LMWH for 10 days. She was advised to continue Flecainide (50 mg tablet twice daily) since the cardioversion. She did not have any recurrences. She was asymptomatic with a normal examination at 10-weeks postnatal cardiology review. A trans-thoracic echocardiograph was normal and Flecainide was stopped. The ethics committee's involvement for this case report was waived as the patient provided an informed consent for publication of the case and all data were anonymized.

## 3 Discussion

New onset maternal AF is rare.^[7–9]^ Potential factors including direct effects of hormones on cardiac electrophysiology, autonomic tone, and hemodynamic perturbations, can provoke arrhythmias in pregnancy, labor, and delivery.^[10]^ Certain other causes are underlying structural heart disease,^[4,5]^ hyperthyroidism,^[11]^ tocolytics (e.g., Terbutaline)^[12]^ and peripartum cardiomyopathy (with a high prevalence of 10%).^[13]^ (Box1: Risk factors for atrial fibrillation in pregnancy)

Matěcha and Riedlbauchová ^[14]^ reported a case of ECV in a pregnant woman with corrected congenital heart disease and new-onset atrial flutter. Murphy et al^[1]^ reported a urinary tract infection as the only potential cause in a 33 weeks pregnant female with dichorionic–diamniotic twins and AF. In the present study, the patient's urinary analysis was normal.

Lee et al^[2]^ reported that pregnant women with AF were older, obese, and mostly white. Our patient was a 37-year-old white female with a body mass index of 34 kg/m^2^. Maternal lone AF happens more often in the third trimester of pregnancy.^[2,4–6]^ Similarly, our patient was at 35 weeks of pregnancy. Sengheiser and Channer^[8]^ presented a unique case where AF complicated 2 successive pregnancies at 41 and 33 weeks of pregnancy, respectively.

Symptoms of palpitations, dizziness, and even syncope, are common in pregnancy but these are rarely associated with significant cardiac arrhythmias.^[7]^ Our patient presented with sudden onset palpitations and mild shortness of breath. Murphy et al^[1]^ also reported a similar case presenting with palpitations.

Clinical assessment and 12-lead ECG investigation are mandatory for the diagnosis of the arrhythmia.^[5,9]^ These patients are thoroughly assessed for cardiovascular risk factors for stroke and thromboembolism. The initial work-up aims to rule out cardiac, as well as extra cardiac etiologies, for example, pulmonary embolism, electrolyte imbalance, pharmacologic effects, etc.^[4,15]^ Echocardiography is recommended in all patients with AF to guide management.^[5,9]^ All possible etiologies need exclusion for the diagnosis of lone AF.^[15,16]^ (see Table 1)

**Table 1:**
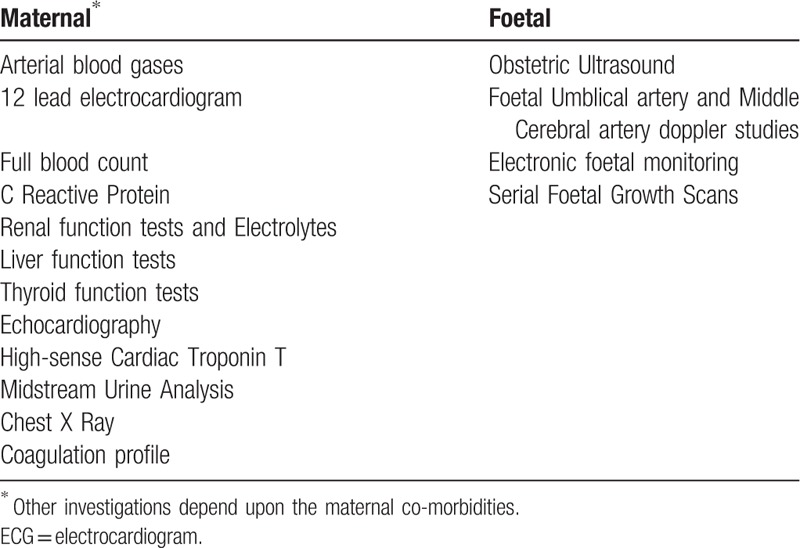
Investigations for maternal atrial fibrillation.

In cases of AF occurring for longer than 48 hours, a transesophageal echocardiograph should be completed to evaluate for atrial thrombus.^[15]^ The patient's hs-cTnT was elevated at 72 ng/L. Literature review emphasizes that level of hs-cTnT is often elevated in patients with AF and it is independently associated with an increased risk of stroke, cardiac death, and major bleeding.^[17]^

These high-risk pregnancies need a multidisciplinary input. The initial supportive measures include left lateral position, 100% oxygen, and early intravenous access.^[18]^ Potential stimulants, such as smoking, caffeine, and alcohol should be eliminated.^[10]^ AF is mainly unprovoked, paroxysmal, and generally stable with spontaneous cadioversion, occurring usually within 24 hours.^[19]^ Vagal nerve stimulation and Valsalva maneuvers are the first line treatments.^[5,20]^

We admit that we should not have given our patient the therapeutic dose of LMWH in the emergency department, before going to operation theatre for ECV because of the potential need for emergency cesarean section for fetal and/or maternal compromise. We were fortunate that no such need arose. Thus, either no or unfractionated heparin infusion would have been a safer choice (advantages of quick and complete reversibility). Literature review suggests that if possible, ECV should be considered within 48 hours of the onset of AF to minimize thromboembolic complications and to avoid the need for anticoagulation.^[7]^ In patients having no risk factors for stroke, anticoagulation is recommended for 4 weeks after cardioversion.^[9]^ Our patient received therapeutic dose of LMWH between 35 and 39 weeks of pregnancy and prophylactic dose of LMWH for 10 days post-cesarean section. LMWH has a better safety profile with fewer side effects .^[7]^ Non-vitamin K antagonist oral anticoagulants should be avoided in pregnancy.

The aim of treatment is maternal stabilization with control of maternal heart rate and rhythm, anticoagulation and to attain fetal maturity in preterm pregnancies. Regarding the pharmacologic therapy for rate control, due to insufficient data to comment on Verapamil and Diltiazem, only use of beta-blockers and/or digoxin is recommended at the lowest dose and for the shortest time required to avoid their potential adverse effects.^[9,10]^ Beta-blockers are associated with fetal growth restriction^[4,9]^; thus necessitating serial fetal growth monitoring in pregnancy. Digoxin may be safe but its use can be problematic due to altered serum levels in pregnancy. In extreme cases of maternal digitalis intoxication, a fetal death has been reported.^[4]^ Rhythm control therapy in pregnant patients with AF has only been reported in few case studies.^[9]^ Flecainide and Sotalol may be safe.^[4]^ Amiodarone is not safe for the fetus.^[7,9,10]^ Judicious use of antiarrthymic and anticoagulant drug therapy in pregnancy is advocated in view of their effects on the developing fetus and maternal physiology.

Depending upon the clinical circumstances, the obstetrician may consider using antenatal corticosteroids for fetal lung maturity in pregnancies, complicated by AF at a gestational age < 39 weeks where chances of cesarean delivery are high, albeit the maternal health is always prioritized.

Maternal AF is associated with increased complications for both the mother and the fetus^[4,9]^ but generally outcome is good.^[19]^ The ultimate feto-maternal prognosis depends on the clinical condition of the mother, other comorbidities, and gestational age of fetus. Our patient delivered a healthy baby 4 weeks after successful ECV at 35 weeks of pregnancy. Those pregnant women who have persistent AF require fetal growth surveillance and antenatal testing.^[16]^ One maternal and fetal death has been reported after attempted cardioversion for AF in a moribund patient with comorbid congestive heart failure. The fetal death was thought to be unrelated to the direct current cardioversion.^[21]^ There were no maternal deaths from AF in MBRRACE-UK Maternal Report 2016^[22]^ (see Table 2).

**Table 2:**
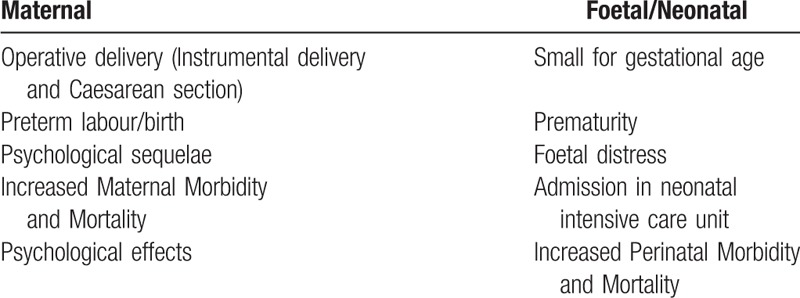
Obstetric complications of maternal atrial fibrillation.

ECV has been performed safely at all stages of pregnancy^[7,8,15]^ and is recommended whenever the risk of ongoing AF is considered high for the mother or the fetus.^[9,15]^ ECV could be performed in stable patients as well to avoid the potential adverse effects of drugs.^[7]^ Energy requirements for ECV are the same as in the nonpregnant women^[20]^ and may vary from 50 to 400 Joule depending upon the physician's choice.^[5]^ Our patient received a single 200 Joule synchronized DC shock. The success rate of ECV in pregnant females is 93.2% after one or more attempts.^[5]^ There are case reports in the literature where radiofrequency catheter ablation has been used in pregnant women.^[23]^ ACC/AHA/HRS guideline 2016^[20]^ for the management of adult patients with supraventricular tachycardia suggests that catheter ablation may be reasonable in pregnant patients with highly symptomatic, recurrent, drug refractory supraventricular tachycardia with efforts toward minimizing radiation exposure. We chose ECV for treating maternal AF because it has been reported safe and more significant concerns about the rate and rhythm controlling drugs.

Propofol can be used for ECV because of its rapid onset, safety, and short action.^[7]^ While doing a cesarean section in a patient with ongoing/previous AF, anesthetic focus is to avoid factors known to produce cardiac ectopy, such as increased sympathetic tone, electrolyte imbalance, and acid–base disturbance.^[24]^ After cardioversion, our patient was started on Flecainide. The 2016 European Society of Cardiology guideline for the management of AF advocates the effectiveness of Flecainide in preventing recurrent AF in patients without ischemic heart disease or heart failure to avoid risk of life-threatening arrhythmias.^[9]^ Our patient did not breast feed her neonate. There is limited data on safety of Flecianide during breastfeeding.^[25]^ Regarding the other antiarrhythmics, the levels of digoxin^[26]^ and verpamil^[27]^ are too low in breast milk to cause any adverse effects in breastfeeding infants.

The factors affecting success of ECV include the type and length of the arrhythmia, cardioversion method, voltage, and type of energy.^[5]^ In spite of the fact that synchronized direct current cardioversion for maternal AF has been reported as being safe,^[5–7]^ and highly effective procedure,^[5]^ its use in pregnancy remains rare. Thus, there have always been concerns regarding its use in pregnant females. The uterus and amniotic fluid are excellent conductors of electricity.^[6]^ During ECV, energy source and trajectory are directed away from the uterus and fetus^[5]^ and the amount of current reaching the fetus is thought to be negligible.^[28]^ Anteroposterior pad placement rather than anterolateral further minimizes this risk.^[29]^ Direct cardioversion may result in sustained uterine contraction, fetal distress, and necessitating emergency cesarean section.^[6]^ Other complications of ECV during pregnancy include premature labor^[29]^ and preterm delivery.^[5]^ Thus, facilities for continuous fetal monitoring^[7,15]^ and cesarean section should be in place during the procedure.^[5–7,15]^ Because of the complexity of the situation, the expectant mother is under stress; thus it is important to explain her the events, the benefits and risks of ECV and seek her consent for ECV and/or cesarean section before the procedure.

## 4 Conclusion

Maternal AF poses a risk to both maternal and fetal well-being and its treatment is a challenge for clinicians. The maternal clinical condition, the presence of underlying heart disease, laboratory tests including 12 lead ECG, echocardiography, hs-cTnT, electronic fetal monitoring, gestational age, and physician's choice generally affect the mode of treatment (Flow Diagram 1: Management of atrial fibrillation in a pregnant female). ECV for AF during pregnancy has been considered as a safe and effective procedure and its known complications include fetal distress and emergency cesarean delivery for maternal and/or fetal compromise. Thus, it is prudent to monitor fetal well-being before and after the procedure and use the LMWH cautiously. In the view of rarity of the condition, maternal and fetal concerns and different clinical experiences and settings, it is difficult to conduct well-designed clinical trials for the treatment of maternal AF.

## Acknowledgments

We would like to thank Professor Dr Naveed Kausar Janjua, Chemistry Department, Quaid-i-Azam University, Islamabad, Pakistan for her continuous support and encouragement. A consent was taken from her to include her name for acknowledgement.

## Author contributions

**Conceptualization:** Nusrat Batool Janjua, Thomas McDonagh.

**Data curation:** Nusrat Batool Janjua, Thomas McDonagh.

**Methodology:** Suhaib Akhtar Birmani.

**Software:** Nusrat Batool Janjua.

**Writing – original draft:** Nusrat Batool Janjua.

**Writing – review & editing:** Nusrat Batool Janjua, Suhaib Akhtar Birmani, Abdul Hameed, Matthew McKernan.

Nusrat Batool Janjua orcid: 0000-0002-2711-0942.

## Supplementary Material

Supplemental Digital Content
