# Supplementary material for: New-onset lone maternal atrial fibrillation: A case report
Source: Medicine (Baltimore). 2020 Feb 14;99(7):e19156. doi: 10.1097/MD.0000000000019156 (PMC7035010; doi:10.1097/MD.0000000000019156)
Supplement: Supplemental Digital Content [file medi-99-e19156-s001.docx]

Box 1: Risk factors for atrial fibrillation in pregnancy

| Infection e.g. urinary tract infection^1^  Old maternal age^2^  Maternal obesity^2^  White race^2^  Second half of pregnancy^2, 4-6,9, 18^  Use of certain drugs e.g. Nifedipine &Terbutaline^19-21^  Peripartum Cardiomyopathy^22^ |
| --- |
